# Heteroaromatic Polyamides with Improved Thermal and Mechanical Properties

**DOI:** 10.3390/polym12081793

**Published:** 2020-08-10

**Authors:** Miriam Trigo-López, Ana M. Sanjuán, Aranzazu Mendía, Asunción Muñoz, Félix C. García, José M. García

**Affiliations:** Departamento de Química, Facultad de Ciencias, Universidad de Burgos, Plaza de Misael Bañuelos s/n, 09001 Burgos, Spain; amsanjuan@ubu.es (A.M.S.); amendia@ubu.es (A.M.); amugnoz@ubu.es (A.M.); fegarcia@ubu.es (F.C.G.)

**Keywords:** aromatic polyamides, aramid, improved properties, mechanical properties, thermal properties, high-performance polymers

## Abstract

We prepared high-performance aromatic copolyamides, containing bithiazole and thiazolo-thiazole groups in their main chain, from aromatic diamines and isophthaloyl chloride, to further improve the prominent thermal behavior and exceptional mechanical properties of commercial aramid fibers. The introduction of these groups leads to aramids with improved strength and moduli compared to commercial *meta*-oriented aromatic polyamides, together with an increase of their thermal performance. Moreover, their solubility, water uptake, and optical properties were evaluated in this work.

## 1. Introduction

Polymers belong to a high-performance family when they have a few outstanding properties and characteristics, and usually need to have, at least, exceptional mechanical and thermal behavior. Aromatic polyamides, usually known in the field with the shorten name aramids, are a representative member of this family because they have specific moduli and strength that outperform conventional materials, such as glass or steel, especially considering their much lower density. Such properties derive from the aromatic nature of the polymer main chain that contains a minimum of 85% of the amide groups directly linked to two aromatic rings, resulting in high chain rigidity and efficient hydrogen bonding (high cohesive energy) [1]. As a result, these materials are used as fibers to prepare innovative protecting materials [2] and composites [3,4]. The marketed aramids include, the *meta*-oriented poly(*m*-phenylene isophthalamide), the *para*-oriented poly(*p*-phenyleneterephthalamide), and the *meta* and *para*-oriented copolymer copoly(*p*-phenylene/3,4′-diphenylether terephthalamide).

Research efforts in the field are oriented in three directions, being the improvement of the properties of marketed aromatic polyamide fibers, or by fully design of the chemical structure to offer innovative properties, or even both, mainly by maintaining the chemical structure, but introducing functionality by copolymerizing conventional monomers with synthetic ones [2]. Herewith, we have followed the third approach by preparing thermally and mechanically improved copolyamides.

The following has to be considered and taken into account to face the improvement of the properties of marketed aromatic polyamides. They show high thermal resistance and keep their mechanical properties even at high temperatures [5] with decomposition temperatures above 400 and 500 °C for *meta*- and *para*-aramids, respectively, as a result of the high bond dissociation energies of C-N and C-C bonds. Also, due to their hydrogen bonding and polymer backbone rigidity, the *T*_g_ values are between 272 and 275 °C for poly(*m*-phenylene isophthalamide) and 295 °C for poly(*p*-phenyleneterephthalamide) [6]. 

Another unique property of commercial aramids, related to their thermal stability, is their low flammability, measured by the LOI (limiting oxygen index). The value for aromatic polyamides is 30–32% and 28–30% for *meta*- and *para*-aramids, respectively. Polymers with flame retardant properties commonly have halogens in their formulation [7,8,9,10].

On the other hand, the introduction of heterocyclic rings in the main chain of aromatic polyamides is known to provide excellent thermal stabilities to the polymers and improve their solubility. Recent advances in this sense include the introduction of phenyl-1,3,5-triazine, or the incorporation of planar dibenzofuran moieties in the polymer backbone, which resulted in polymers high *T*_g_s and excellent thermal stabilities [11,12].

In relation to the mechanical properties, recent efforts towards their improvement include the use of phthalazinone moieties [13], fluorine-containing monomers [6,14], cyano groups [15], bulky xanthene groups [8] or 4-aryl-2,6-diphenylpyridine moieties [16], in the polymer structure. The crosslinking of polymeric chains in aramids leads to polymers with improved thermal and mechanical properties, as demonstrated in some works [17,18]. Aromatic bithiazoles are well-studied compounds and are commonly used, regarding their biological properties, as antivirals against hepatitis B or HIV [19,20], and also in polymers as fluorescent chemosensors, and in polymer complexes with magnetic behavior [21], and in photovoltaics and thin-film transistors [22,23,24]. Also, thiazolo [4,5-d]thiazoles are recently studied as new domains for semiconductors and potential optoelectronic applications, showing intramolecular charge transfer [25,26]. Moreover, the intermolecular interactions between S and N atoms are relevant in incrementing the interchain interactions increasing the cohesive energy and, accordingly, in the thermal and mechanical performance of polymers having this group in their main chain [27].

## 2. Experimental Procedures

### 2.1. Materials

We used the chemicals and solvents without further purification unless indicated. 4-nitrobenzaldehyde (for synthesis, Merck, Darmstadt, Germany), 4-nitroacetophenone (98%, Alfa Aesar, Kandel, Germany), Br_2_ (99.8%, Alfa Aesar), dithiooxamide (98%, Alfa Aesar), sulfuric acid (95%, VWR-Prolabo, Fonteney-sous-bois, France) diethyl ether (VWR-Prolabo), ethanol (99.9%, VWR-Prolabo), ethyl acetate (VWR-Prolabo), dimethyl sulfoxide (DMSO) (99%, VWR-Prolabo), SnCl_2_·2H_2_O (98%, Alfa Aesar), benzoyl chloride (99%, Sigma-Aldrich, St. Louis, MO, USA), methanol (99.8%, Sigma-Aldrich), and deuterated dimethyl sulfoxide (DMSO-*d*_6_) (99.80D, VWR-Prolabo), *N*,*N*-dimethylacetamide (DMA) (99%, Sigma-Aldrich. Vacuum distilled twice over phosphorous pentoxide and immediately stored over 4 Å molecular sieves). *m*-phenylenediamine (MPD) (>99%; Sigma-Aldrich. Purified by double vacuum sublimation). Isophthaloyl dichloride (ICL) (>99%, Sigma-Aldrich. Purified by double crystallization upon dry heptane (99.9%, VWR-Prolabo)).

### 2.2. Measurements

Nuclear Magnetic Resonance spectra (NMR) were obtained in a Varian Inova 400 spectrometer (Varian, Palo Alto, CA, USA) functioning at 400 (^1^H) and 100.6 (^13^C) MHz, using DMSO-*d*_6_ as solvent. Infrared spectra (FTIR) were registered with a Jasco Spectrometer FT/IR-4200 FTIR (Jasco, Victoria, Canada) with an ATR-PRO410-S single reflection accessory. Fluorescence spectroscopy was carried out using a Hitachi spectrophotometer (F-7000 fluorescence, (Hitachi, Tokyo, Japan)), and Ultraviolet/Visible (UV/Vis) spectroscopy was performed in a Hitachi spectrophotometer (U-3900, (Hitachi, Tokyo, Japan)). High-resolution electrospray ionization mass spectra (ESI-HRMS) were obtained on a coupled liquid chromatographer-mass spectrometer (Agilent Infinity 1260-Agilent 6545, (Agilent, Santa Clara, CA. USA)).

Thermal analysis was carried out by thermogravimetric measurements (TGA, TA Instrument Q50 TGA analyzer, (TA Instruments, New Castle, DE, USA)) using a 5 mg sample under a nitrogen or oxygen atmosphere at a 10 °C/min scan rate. The LOI was calculated using the char yield mass percentage (CR) at 800 °C (obtained from the TGA measurements under a nitrogen atmosphere from the equation LOI = 17.5 + 0.4 × CR (Van Krevelen, experimental equation [28]).

Differential Scanning Calorimetry (DSC) experiments were carried out to determine the thermal transitions of the copolyamides (DSC Q200, TA Instruments, New Castle, DE, USA); N_2_ atmosphere, flow rate 50 mL/min, about 15 mg of sample.). The thermal analysis of the samples was performed as follows: First, a cycle was run to the samples to erase the thermal history consisting of 5 min of stabilization at 30 °C, then, the samples were heated to 400 °C at a rate of 15 °C per minute and stabilized for 5 min at that temperature, then cooled down to −80 °C. The second scan of up to 400 °C was performed at 15 °C per minute. 

The polymer solubility was evaluated with 10 mg of the polymer and 1 mL of the solvent and stirring for 24 h at 20 °C. If a homogeneous solution is observed, the polymer is considered soluble at room temperature; otherwise, the system is heated to reflux for two hours, and the polymer is considered soluble on heating if a homogenous solution is formed. If no homogeneous solution is observed after that, the polymer is considered insoluble. 

The polymer’s inherent viscosity, *η_inh_*, was determined with a Canon-Fenske viscometer from a 0.5 g/dL polymer concentration using sulfuric acid (95%) as the solvent at 25 ± 0.1 °C. The intrinsic viscosity, [*ղ*], was obtained measuring the *ղ_inh_* at different polymer concentrations (0.5, 0.3, 0.1 and 0.05 g/dL) using the same solvent at 25 ± 0.1 °C and extrapolating to zero concentration. The number overage molecular mass (M_w_) was estimated from [*ղ*] using the viscometric equation [*η*] = κΜ_ω_^α^ with the constants of poly(*m*-phenylene isophthalamide) (κ = 0.00013 dL/g; α = 0.84) [29].

Water uptake measurements were performed gravimetrically. Dried samples (about 23 mg, dried at 60 °C for 24 h under vacuum and phosphorus pentoxide) were placed in a closed box at 20 °C having a relative humidity of 65% (provided by a saturated aqueous solution of NaNO_2_). The samples were weighed periodically until obtaining a constant mass. The water molecules per structural unit are calculated considering the weight of the structural unit and the water uptake. In copolymers, a hypothetical structural unit is calculated by considering the relative weight of the two structural units that comprise the copolyamides.

Films from copolyamides **CP1** and **CP2** were prepared by *casting* in DMA (solution-evaporation) with about 7% by polymer mass. Measurements of mechanical properties were performed with these untreated films with Shimazdu equipment (EZ Test Compact Table-Top Universal Tester, Shimadzu, Kyoto, Japan). The films were cut in 5 × 40 mm strips, and the tests were performed with five samples of each film, averaging the data, with the following measurement conditions: 5 mm/min extension rate, 9.44 mm gauge length. 

The wide-angle X-ray diffraction patterns were acquired from powdered polyamides (2 h in a SPEX SamplePrep 8000M Mill) using a Bruker D8 Discover Davinci X-ray diffractometer (40 kV, Bruker, Billlerica, MA. USA), Co as the radiation source, scan step size of 0.05 with a scan step time of 2 s for **CP1** and 3 s for **CP2**).

### 2.3. Synthesis of Intermediates, Monomers, and Models

#### 2.3.1. Synthesis of Diamine D1 and Model Polyamide M1

**Synthesis of 2-bromo-1-(4-nitrophenyl)ethan-1-one (1)**: Br_2_ (100 mmol, 5.13 mL) was slowly added drop by drop to a solution of 4-nitroacetophenone (100 mmol, 16.5 g) in diethyl ether (300 mL) at 0 °C, and then stirred at room temperature for 2 h. The solvent was evaporated under reduced pressure, and a solid was obtained (99% yield). ^1^H NMR (400 MHz, DMSO-*d*_6_, δ) = 8.16 (d, *J* = 9 Hz, 2H, Ph), 7.98 (d, *J* = 9.8 Hz, 2H, Ph), 4.30 (s, 2H, CH_2_). HRMS (ESI) calculated for ([C_8_H_6_BrNO_3_]+H)^+^: Not found. FT-IR [wavenumbers (cm^−1^)]: ʋ_C=O_: 1698, ʋ_NO2 (As, S)_: 1513, 1340. 

 

**Synthesis of 4,4′-bis(4-nitrophenyl)-2,2’-bithiazole (2)**: In a reduced pressure rounded-bottom flask dithiooxamide (10.23 mmol, 1.23 g) was added to a solution of 2-bromo-1-(4-nitrophenyl)ethan-1-one **1** (20.5 mmol, 5 g) in ethanol (100 mL) and was stirred at 78 °C overnight. The mixture was cooled to room temperature, and the brown solid formed was filtered and washed with methanol (86% yield). ^1^H NMR (400 MHz, DMSO-*d*_6_, δ) = 8.72 (s, 2H, =CH), 8.41–8.27 (m, 8H, Ph). ^13^C NMR was not possible due to the insolubility of the compound. HRMS (ESI) calculated for ([C_18_H_10_N_4_O_4_S_2_]+H)^+^: 411.0216; found: 411.0213. FT-IR [wavenumbers (cm^−1^)]: ʋ_Ar C-H_: 3115, ʋ_C-N_: 1595, ʋ_NO2 (As, S)_: 1508, 1336. 

 

**Synthesis of the monomer,4,4′-([2,2′-bithiazole]-4,4′diyl)dianiline (D1)**: To a solution of 4,4′-bis(4-nitrophenyl)-2,2′-bithiazole **2** (16.5 mmol, 6.95 g) in ethanol (55 mL) was added SnCl_2_·2H_2_O (165 mmol, 38.08 g) in a reduced pressure rounded-bottom flask and heated at 70 °C for 4 h. The mixture was poured into a combination of ice-water, and the pH was adjusted to 8–9 with NaHCO_3_. Ethyl acetate was used to extract the resulting mixture (3 times), and the combined organic layers were dried over Na_2_SO_4_ and concentrated at reduced pressure. The remaining residue was used without further purification (55% yield). ^1^H NMR (400 MHz, DMSO-*d*_6_, δ) = 7.87 (s, 2H, =CH), 7.70 (d, 4H, *J* = 8.6 Hz, Ph), 6.66 (d, 4H, *J* = 8.6 Hz, Ph), 5.36 (s, 4H, NH_2_). ^13^C NMR (100.6 MHz, DMSO-*d*_6_, δ) = 160.0, 156.6, 149.2, 127.2, 121.4, 113.8, 111.9. HRMS (ESI) calculated for ([C_18_H_14_N_4_S_2_]+H)^+^: 351.0733; found: 351.0739. FT-IR [wavenumbers (cm^−1^)]: ʋ_NH2_: 3412, 3324, ʋ_Ar C-H_: 3095, ʋ_C-N_: 1601.

 

**Synthesis of *N*,*N’*-([2,2′-bithiazole]-4,4′diylbis(4,1-phenylene))dibenzamide (M1)**: Benzoyl chloride (3.43 mmol, 298.75 µL) was added portionwise to a solution of the previously prepared diamine **D1** (1.43 mmol, 0.5 g) in *N*,*N*-dimethylacetamide (5 mL) at 0 °C under nitrogen. After 30 min the resulting mixture was stirred at room temperature for an additional 4 h. Then, the solution was poured into distilled water forming a yellowish solid which was filtered and washed with water yielding 81%. ^1^H NMR (400 MHz, DMSO-*d*_6_, δ) = 10.42 (s, 2H, NH); 8.29 (s, 2H, CH); 8.12–7.85 (m, 12H, Ph); 7.66–7.49 (m, 6H, Ph). ^13^C NMR (100.6 MHz, DMSO-*d*_6_, δ) = 165.6; 160.4, 155.3, 139.5, 134.8, 131.7, 128.70, 128.41, 127.70, 126.52, 120.45, 115.89. HRMS (ESI) calculated for ([C_32_H_22_N_4_O_2_S_2_]+H)^+^: 559.1257; found: 559.1254. FT-IR [wavenumbers (cm^−1^)]: ʋ_NH_: 3264, ʋ_Ar C-H_: 3108, ʋ_C=O_: 1648.

#### 2.3.2. Synthesis of Diamine D2 and Model Polyamide M2

**Synthesis of 2,5-bis(4-nitrophenyl)thiazolo[5,4-*d*]thiazole (3)**: Dithiooxamide (66.17 mmol, 7.95 g) was added to a suspension of 4-nitrobenzaldehyde (132.34 mmol, 20 g) in nitrobenzene (130 mL) and heated at 130 °C overnight. The resulting mixture was cooled to room temperature, and diethyl ether was added, forming a red solid which was filtered and washed with Et_2_O (61%) and employed in the next step without further purification. Due to the insolubility of the compound, NMR and HRMS were not possible to do. FT-IR [wavenumbers (cm^−1^)]: ʋ_NO2 (As, S)_: 1513, 1340.

 

**Synthesis of the monomer 4,4′-(thiazolo[5,4-*d*]thiazole-2,5-diyl)dianiline (D2)**: To a solution of 2,5-bis(4-nitrophenyl)thiazolo[5,4-*d*]thiazole **3** (40.6 mmol, 15.6 g) in ethanol (200 mL) was added SnCl_2_·2H_2_O (406 mmol, 91.6 g) in a reduced pressure rounded-bottom flask and heated at 70 °C for 4 h. The mixture was poured into a combination of ice-water, and the pH was adjusted to 8–9 with NaHCO_3_. Ethyl acetate was used to extract the resulting mixture (3 times), and the combined organic layers were dried over Na_2_SO_4_ and concentrated at reduced pressure. The remaining residue was pure and was used without further purification (28% yield). ^1^H NMR (400 MHz, DMSO-*d*_6_, δ) = 7.65 (d, 4 H, J = 8.6 Hz, Ph), 6.65 (d, 4 H, J = 8.6 Hz, Ph), 5.83 (s, 4H, NH). ^13^C NMR (100.6 MHz, DMSO-*d*_6_, δ) = 168.75, 152.04, 148.31, 127.86, 121.24, 114.13. HRMS (ESI) calculated for ([C_16_H_12_N_4_S_2_]+H)^+^: 325.0576; found: 325.0578. FT-IR [wavenumbers (cm^−1^)]: ʋ_NH2_: 3452, 3324, ʋ_Ar C-H_: 3311, ʋ_C-N_: 1598.

 

**Synthesis of *N*,*N’*-(thiazolo[5,4-*d*]thiazole-2,5-diylbis(4,1-phenylene))dibenzamide (M2)**: Benzoyl chloride (3.7 mmol, 319.45 µL) was added portion-wise to a solution of the previously prepared diamine **D2** (1.54 mmol, 0.5 g) in *N*,*N*-dimethylacetamide (5 mL) at 0 °C under nitrogen atmosphere. After 30 min, the resulting mixture was stirred at room temperature for another 4 h. Then, the solution was poured into distilled water, forming a brown solid that was filtered and washed with water (yield: 78%). ^1^H NMR (400 MHz, DMSO-*d*_6_, δ) = 10.42 (s, 2H, NH), 8.20–7.86 (m, 12H, Ph), 7.69–7.47 (m, 6H, Ph). ^13^C NMR (100.6 MHz, DMSO-*d*_6_, δ) = Not possible due to the compound’s solubility. HRMS (ESI) calculated for ([C_30_H_20_N_4_O_2_S_2_]+Na)^+^: 555.0920; found: 555.0917. FT-IR [wavenumbers (cm^−1^)]: ʋ_NH_: 3340, ʋ_C-N_: 1523.

#### 2.3.3. Synthesis of Copolyamides CP1 and CP2

**Synthesis of copolyamide CP1**: Under nitrogen atmosphere in a three-necked flask with a mechanical stirrer was added *N*,*N*-dimethylacetamide (4.5 mL) at room temperature. Then, diamine **D1** (0.443 mmol, 0.155 g) and *m*-phenylenediamine (3.989 mmol, 0.431 g) were added. The mixture was cooled to 0 °C, and isophthaloyl dichloride (4.43 mmol, 0.9 g) was added portion-wise. The reaction was reacted at 0 °C for 30 min and then heat to room temperature for another 4 h. The solution was poured into distilled water rendering a swollen white precipitate that was filtered and washed with acetone (quantitative yield).

 

**Synthesis of copolyamide CP2**: Under nitrogen atmosphere in a three-necked flask with a mechanical stirrer was added *N*,*N*-dimethylacetamide (4.5 mL) at room temperature. Then, diamine **D2** (0.443 mmol, 0.144 g) and *m*-phenylenediamine (3.989 mmol, 0.431 g) were added. The mixture was cooled to 0 °C, and isophthaloyl dichloride (4.43 mmol, 0.9 g) was added portion-wise. The reaction was reacted at 0 °C for 30 min and then heat at 50 °C for another 4 h. The solution was cooled to room temperature and poured into distilled water, rendering a yellow swollen polymer precipitate, filtered and washed with acetone (quantitative yield).

## 3. Results and Discussion

The technological world demands materials with higher mechanical and thermal performance. Accordingly, we describe herein the design, synthesis, characterization and evaluation of the properties of high-performance polymers for advanced applications, such as civil protection or aeronautics. We describe aromatic copolyamides with 10% of heteroaromatic groups (bithiazol or thiazolo[4,5-d]thiazol, **CP1** and **CP2**, respectively) in the main chain of the polymer, which resulted in polymers with improved thermal stability and mechanical properties. It is known that aramids with small modifications in the structural units compared to poly(*m*-phenylene isophthalamide) can provide enhanced characteristics to the polymer without penalizing their outstanding performance [18,30].

To increase the thermal and mechanical characteristics of high-performance aromatic polyamides we have included in the aromatic main structure bithiazoles and thiazolo-thiazole moieties. These groups provide strong intermolecular interactions between S and N atoms, thus increasing the interchain interaction resulting in more efficient aggregation, higher cohesive energy, and higher mechanical and thermal properties [26].

To compare their performance, some of these synthesized polyamides’ properties were compared with the properties of commercial poly(*m*-phenylene isophthalamide) synthesized as described in previous work [31] and the same conditions as **CP1** and **CP2**.

### 3.1. Synthesis and Characterization of the Monomer, the Model and the Polymers

**Scheme 1:**
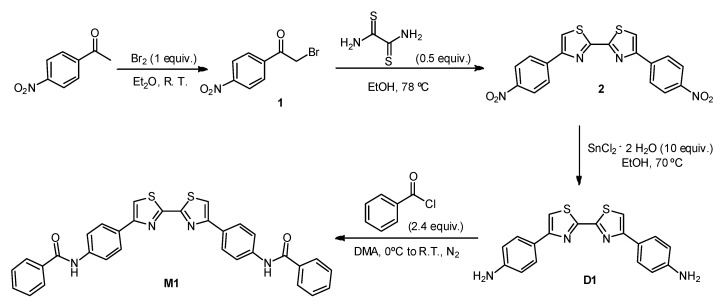
Synthesis of diamine **D1** and model polyamide **M1**.

**Scheme 2:**
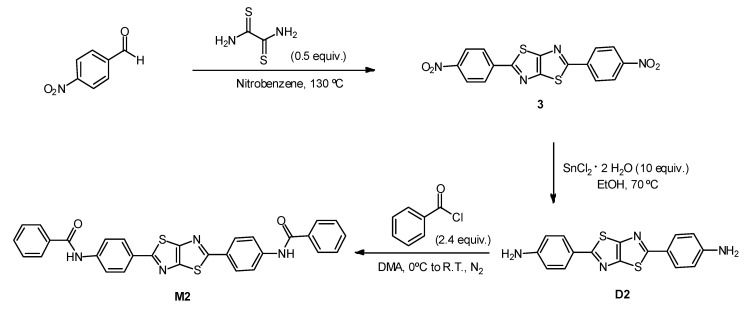
Synthesis of diamine **D2** and model polyamide **M2**.

Diamines **D1** and **D2** were prepared following easy and are well-accepted reactions from the organic chemistry point of view (Scheme 1 and Scheme 2). The chemicals used in the synthesis of the monomers are commercial and cheap, and the reactions proceed in relatively good yields and with high purity, as verified by the NMR spectra. The chemical structure of all the intermediates and the monomers was confirmed by FTIR, ^1^H and ^13^C, and HRMS. However, due to some of the intermediates’ insolubility, it was not possible to perform the ^13^C NMR in some cases, or even the HRMS for intermediates **1** and **3**. Still, this was not a limiting fact since it was possible to characterize the rest of intermediates, monomers and model polyamides in the subsequent steps, confirming the products’ structure.

Model polyamides **M1** and **M2** were synthesized before the polymer synthesis using polymerization conditions (Scheme 1 and Scheme 2). This strategy is recommended in the preparation of aramids to test the viability of the polyamide synthesis by characterizing the models, discard byproducts that would impair the preparation of the polymer, and thus their performance. Also, aramids are highly insoluble, and the preparation of model polyamides facilitates their characterization.

**Scheme 3:**
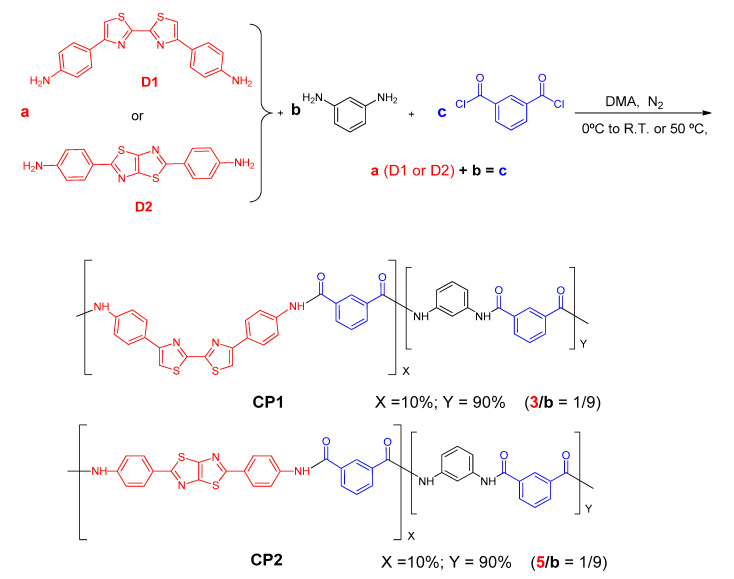
Synthesis of copolyamides **CP1** and **CP2**.

Copolyamide **CP1** was prepared following the conventional procedure from *m*-phenylene diamine and isophthaloyl dichloride (commercial monomers), and diamine **D1** in DMA at 0 °C and then heating at room temperature (Scheme 3). **CP2** was prepared similarly from the two commercial monomers and diamine **D2**, but this time it was necessary to heat the mixture to 50 °C due to the insolubility of the diamine. The structure of the polyamides is shown in Scheme 3. Their inherent viscosities and molecular masses are depicted in Table 1. ^1^H and ^13^C NMR spectra are consistent with the copolyamides structure, and show a high degree of polycondensation, confirmed by the molecular mass of the polyamides (3.4 × 10^4^ and 3.3 × 10^4^ for **CP1** and **CP2**, respectively) obtained from the values of intrinsic viscosities using the Mark-Houwink-Sakurada (MKS) equation [28].

We prepared polymer films from these two copolyamides to evaluate their mechanical and optical performance following a *casting* procedure, using about 7% (*mass*/*volume*) solutions of the copolyamides in DMA.

### 3.2. Properties of the Polymers

#### 3.2.1. Water Uptake and Solubility

Table 2 shows the solubilities of the polyamides and the models. Aramids are soluble only in polar aprotic solvents, and **CP1** and **CP2** show low solubility (lower than the solubility of commercial poly(*m*-phenylene isophthalamide)) in these solvents, due to the rigid and ordered structure derived from the heteroaromatic rings of the main chain coming from the N and S interactions of the heteroaromatic moieties of the main chain [26]. Also, model polyamides show low solubility in polar aprotic solvents and are insoluble in other organic solvents.

The polar groups of an organic molecule determine its hydrophilicity or hydrophobicity. The amide linkage of aromatic polyamides is highly polar, and thus it absorbs water from the environment. Water sorption in aramids enhances some properties while prejudices others. It weakens the amide-amide interactions, lowers the thermal transitions, the mechanical and thermal properties, and impairs electrical insulation. On the other hand, the high water uptake is desirable for applications such as membranes for water desalination. Commercial poly(*m*-phenylene isophthalamide) absorbs 5.2% of water, and **CP1** and **CP2** show comparable water sorption, of around 7% (Table 2). Considering the number of water molecules of water per structural unit, the values of **CP1** and **CP2** are slightly lower than the 1.2 molecules of water of poly(*m*-phenylene isophthalamide).

#### 3.2.2. Mechanical Properties of the Copolyamides

The results from the measurements of the mechanical properties of the films from **CP1** and **CP2** were higher than those found for the lab-made poly(*m*-phenylene isophthalamide) films prepared by casting in the same conditions (Table 3 and Supplementary Materials, Section S6). This improvement in the moduli and the tensile strength probably originates from the additional interchain interactions derived from the presence of S and N atoms in the structures of the new monomers, as previously commented. At this point, it should be considered that the mechanical properties have been calculated using films obtained by casting without post-thermal and mechanical treatment. However, the differences with commercial aramids are relevant because we have obtained and measured the properties with respect to the reference aramid in the same conditions.

#### 3.2.3. Thermal Properties of the Copolyamides

The thermal performance of the polyamides was tested by DSC and TGA analyses. The TGA measurement results are depicted in Table 4 and Supplementary Materials, Section S7). 5% and 10% mass loss temperatures (*T*_5_ and *T*_10_) of the copolyamides are over 445 °C, and 469 °C, respectively, both in nitrogen and in air. These are significantly higher than that of commercial poly(*m*-phenylene isophthalamide) (419 °C and 432 °C).

The char yields are also relevant parameters. These were over 57% at 800 °C in nitrogen. The char yield at a given temperature is associated with the polymer’s thermal behavior, and it is used to evaluate the limiting oxygen index (LOI, Van Krevelen equation [27]). The polyamides showed LOI values over 40, meaning that they can be considered self-extinguishing polymers, as air contains approximately 21% of oxygen. The LOI values of **CP1** and **CP2** (40 and 41, respectively) are also improved compared to commercial poly(*m*-phenylene isophthalamide) (LOI value of 38).

On the other hand, the *T*_g_ is higher for the new copolyamides than for the commercial poly(*m*-phenylene isophthalamide) (Table 4), showing the positive impact of the heterocycle in the structural unit, the rigidity of the chain derived from the rigidity of the monomer in the case of **CP2**, and the extra interchain interactions (amide/heterocycle, heterocycle/heterocycle) compared to conventional poly(*m*-phenylene isophthalamide).

#### 3.2.4. Wide Angle Powder X-ray Scattering

**Figure 1:**
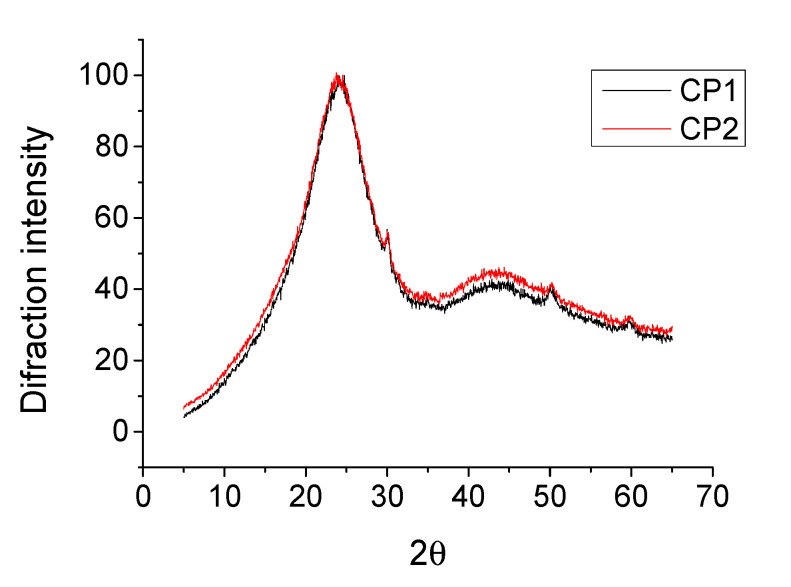
Powder X-ray scattering of **CP1** and **CP2**.

The crystallinity of both polyamides was tested, and they displayed an amorphous pattern (Figure 1). The average molecular interchain spacing (<*R*>) can be calculated [32] from the maximum (θ*_max_*) of the x-ray diffraction for amorphous polymers, using the equation <*R*> = 5/8 (λ/sin θ*_max_*). The average distance of the polymer chains in the amorphous region was estimated to be 4.43 Å in the first amorphous region. In contrast, it is 4.69 Å for commercial poly(*m*-phenylene isophthalamide) (calculated from the work of Lee et al. Figure 1 [33]). The difference in distances agrees with the proposed higher interchain interaction provided by the heteroaromatic moieties in the newly reported aramids.

#### 3.2.5. Optical Properties

##### Luminescence Properties

**Figure 2:**
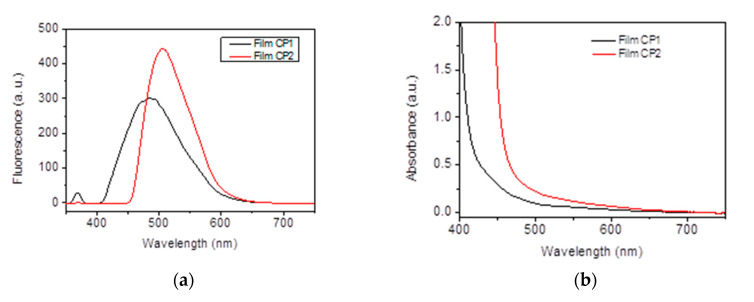
(**a**) Emission spectra of films of **CP1** and **CP2** with under an excitation wavelength of λ_ex_ = 370 nm. (maximum emission λ_em_ Film CP1 = 485 nm. λ_em_ Film CP2 = 505 nm). (**b**) Absorbance spectra of **CP1** and **CP2** films.

The photoluminescence properties of the films obtained from the copolyamides were tested. Fluorescence spectra of **CP1** and **CP2** films were obtained applying different excitation wavelengths to test their emission behavior. Emission fluorescence was observed when the films were irradiated at 370 nm, showing maximum emission bands at 485 nm for **CP1** and 505 nm for **CP2**. Figure 2 shows the fluorescence emission (a) and absorption spectra (b) of **CP1** and **CP2** films.

The quantum yield was calculated for model polyamides **M1** and **M2** in DMA solution, using a quinine solution in 0.05 M sulfuric acid as the fluorescence pattern, and an excitation wavelength of 370 nm. The results obtained show that model polyamide **M2** has a considerably higher quantum yield (Ø_370_ = 0.225) than **M1** (Ø_370_ = 0.028). The quantum yield of **M2** was higher than other values reported for some fluorescent aromatic polyamides for sensory applications [34,35]. However, the quantum yields achieved are still too low for fluorescent materials to be used in light-emitting applications [2,36].

##### CIE 1931

**Figure 3:**
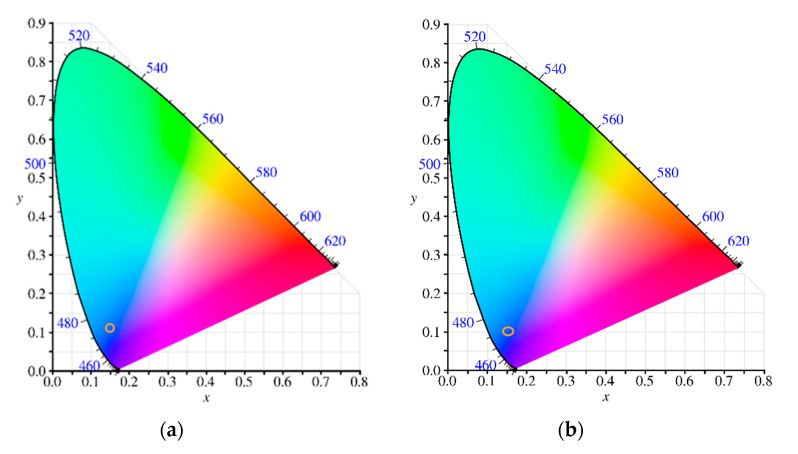
CIE chromaticity coordinates of **a**) **M1** (x = 0.15, y = 0.12) and **b**) **M2** (x = 0.15, y = 0.10). λ_ex_ = 370 nm.

The color spectrum visible to the average human, the perceived colors, can be represented following a numerical model, the CIE color model, where the coordinates *x* and *y* specify the colors in practice. So they were evaluated for the model polyamides. Figure 3 shows the CIE chromaticity coordinates of **M1** and **M2**, calculated from the fluorescence spectra using CIE 1931 color-matching functions (*x* and *y*) [37]. The CIE coordinates were: **M1** (x,y; λ_exc_): (0.15, 0.12; 370 nm); **M2** (x,y; λ_exc_): (0.15, 0.10; 370 nm) [38].

## 4. Conclusions

In short, we have successfully prepared two fluorescent copolyamides containing bithiazole and thiazolo-thiazole groups in their main chain, obtaining aramids with improved thermal and mechanical properties compared to commercial aramids, as a result of the additional interchain interactions. Therefore, the modification of the structure of commercial aramids by introducing heteroaromatic moieties brings new characteristics to commercial high-performance polymers, opening new application fields. The newly synthesized aramids could be used for advanced applications, such as biomedicine, fluorescent chemosensors, photovoltaics or optoelectronic devices, and advanced fibers and composites.

## Figures and Tables

**Table 1:** Inherent viscosity, intrinsic viscosity, and molecular mass of polymers **CP1** and **CP2**.

Copolymer	*η_inh_*^a^ (dL/g)	[*η*] ^b^ (dL/g)	M_w_^c^
**CP1**	0.72	0.83	3.4 × 10^4^
**CP2**	0.80	0.82	3.3 × 10^4^

^a^ 0.5 g/dL solution in H_2_SO_4_. ^b^ 0.5, 0.3, 0.1 and 0.05 g/dL solution in H_2_SO_4_. ^c^ From [*η*] and the MKS equation and viscometric constants of poly(*m*-phenylene isophthalamide (κ = 0.00013 dL/g; α = 0.84) [29].

**Table 2:** Water uptake and solubility of aromatic polyamides and the models.

Model or Polymer	Water Uptake ^a^		Solubility ^b^				
	Mass (%)	Water Molecules Per Structural Unit ^c^	DMA	DMF	DMSO	NMP	EtOH, THF, Acetone, CH_2_Cl_2_, CHCl_3_
**M1**		−−	++	+	+	++	−
**M2**		−−	+−	+−	+	+	−
**CP1**	7.1	0.98	++	++	++	++	−
**CP2**	7.2	1.02	++	+−	+	++	−
Reference ^d^	5.2	1.2	++	++	++	++	−

^a^ RH = 65%, T = 20 °C. ^b^ 10 mg of polymer/1 mL of solvent; ++ = soluble at room temperature; + = soluble on heating; +− = partially soluble; − = insoluble. ^c^ The structural unit is hypothetical in copolymers. It is calculated using the mass and feed ration conforming the polyamide. ^d^ Poly(*m*-phenylene isophthalamide).

**Table 3:** Mechanical properties of **CP1** and **CP2** films.

Polymer	Young’s Modulus (GPa)	Tensile Strength (MPa)
**CP1**	2.2	77
**CP2 ^b^**	1.9	70
Reference ^a^	1.4	63

^a^ Poly(*m*-phenylene isophthalamide). ^b^ **CP2** mechanical properties values are lower than expected probably because of the difficulties in preparing the films by casting due to the lower solubility of the polymer compared to **CP1**.

**Table 4:** Thermal TGA data of the polymers in an inert (N_2_) and an oxidizing (synthetic air) atmosphere.

Polymer	Under Nitrogen			Under Oxygen (Synthetic Air)			LOI ^c^	*T_g_* (°C)
	T_5_^a^ (°C)	T_10_^b^ (°C)	CR (%)	T_5_^a^ (°C)	T_10_^b^ (°C)	CR (%)		
**CP1**	**449**	470	57	451	483	0.7	40	280
**CP2**	445	469	58	446	478	0.8	41	286
Reference ^d^	432	452	50	419	445	9	38	273

^a^ 5% Mass loss (*T*_5_), ^b^ 10% mass loss (*T*_10_). ^c^ Limiting oxygen index, calculated from the TGA data at 800 °C. ^d^ Poly(*m*-phenylene isophthalamide), commercial aramid, synthesized in the same conditions as **CP1** and **CP2**.

## Supplementary Materials

The following are available online at https://www.mdpi.com/2073-4360/12/8/1793/s1, Supplementary information contains the FTIR, ^1^H NMR, and ^13^C NMR of the intermediates, monomers, model polyamides and polymers, and thermal and mechanical characterization of the polymers.
