# Supplementary material for: Heteroaromatic Polyamides with Improved Thermal and Mechanical Properties
Source: Polymers (Basel). 2020 Aug 10;12(8):1793. doi: 10.3390/polym12081793 (PMC7463973; doi:10.3390/polym12081793)
Supplement: Supplementary file 1 [file polymers-12-01793-s001.pdf]

Supplementary Information

Heteroaromatic polyamides with improved thermal and mechanical properties

Miriam Trigo-López,* Ana M. Sanjuán, Aranzazu Mendía, Asunción Muñoz, Félix C. García, José M. García*

Departamento de Química, Facultad de Ciencias, Universidad de Burgos, Plaza de Misael Bañuelos s/n, 09001 Burgos, Spain. E-mail: jmiguel@ubu.es (JMG), mtrigo@ubu.es (MTL)

Table of Contents

S1. Characterization of diamine **D1** and intermediates.......... 2
S2. Characterization of diamine **D2** and intermediates.......... 4
S3. Characterization of model polyamide **M1**.......... 6
S4. Characterization of model polyamide **M2**.......... 6
S5. Characterization of polymers **CP1**, **CP2** and reference polyamide.......... 8
S6. Mechanical properties of polyamide films 11
S7. Thermogravimetric analysis of polyamides.......... 12

S1. Synthesis and characterization of diamine D1 and intermediates

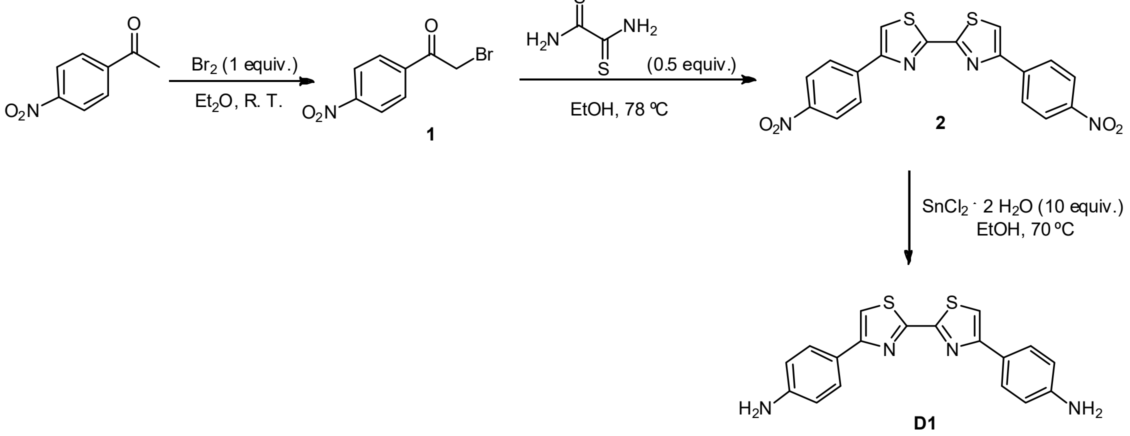

Characterization of 2-bromo-1-(4-nitrophenyl)ethan-1-one (1):

a)

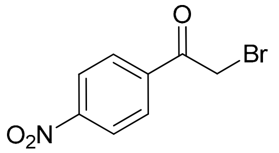

1

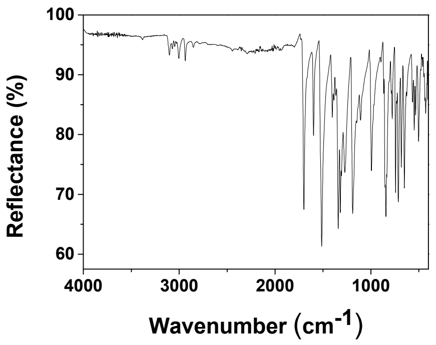

b)

c)

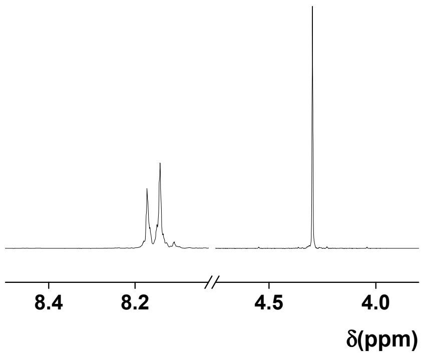

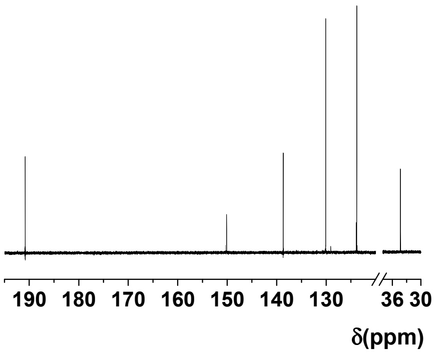

Figure S1. Characterization of **1** by (a) FTIR, (b) ^{1}H NMR, and (c) ^{13}C NMR.

Characterization of 4,4’-bis(4-nitrophenyl)-2,2’-bithiazole (2):

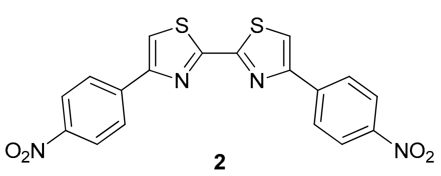

a)

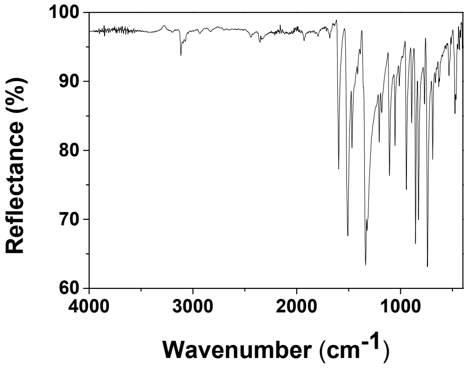

b)

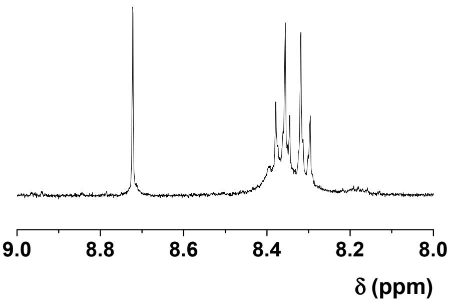

Figure S2. Characterization of **2** by (a) FTIR, (b) ^{1}H NMR.

Characterization of the monomer 4,4’-([2,2’-bithiazole]-4,4’diyl)dianiline (D1):

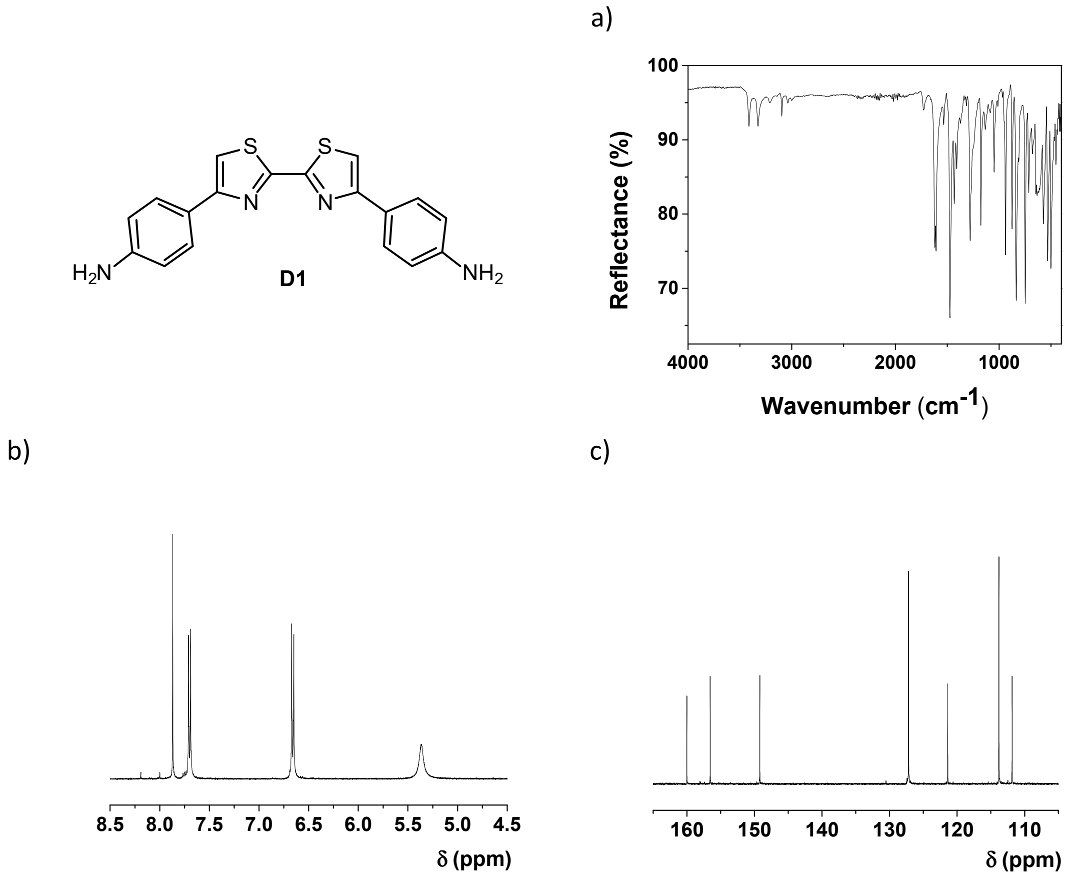

Figure S3. Characterization of **D1** by (a) FTIR, (b) ^{1}H NMR, and (c) ^{13}C NMR.

S2. Characterization of diamine D2 and intermediate

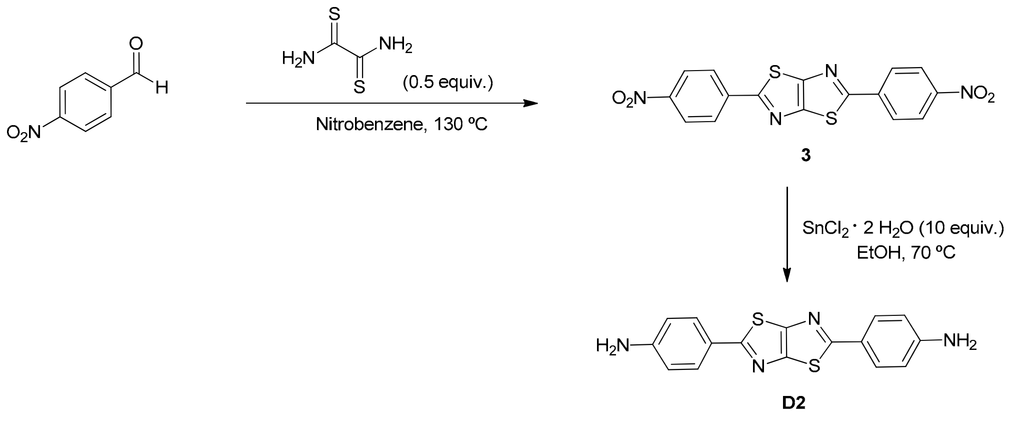

Characterization of 2,5-bis(4-nitrophenyl)thiazolo[5,4-d]thiazole (3):

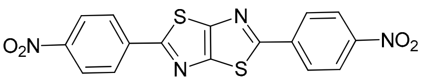

3

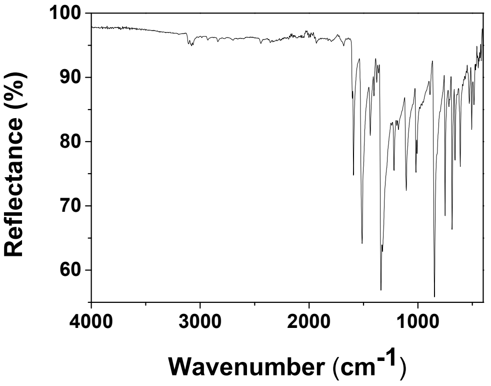

Figure S4. Characterization of **3** by FTIR.

Characterization of the monomer 4,4'-(thiazolo[5,4-d]thiazole-2,5-diyl)dianiline (D2):

a)

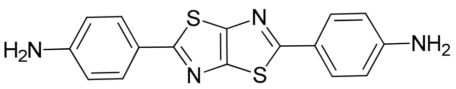

D2

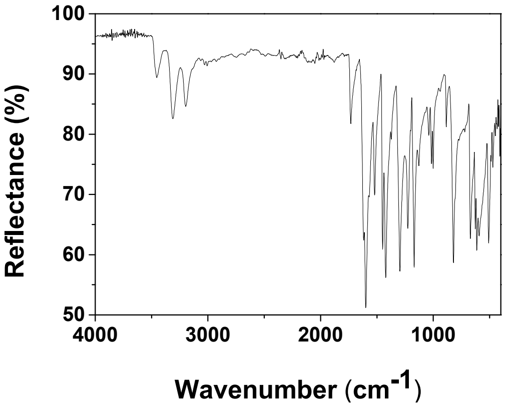

b)

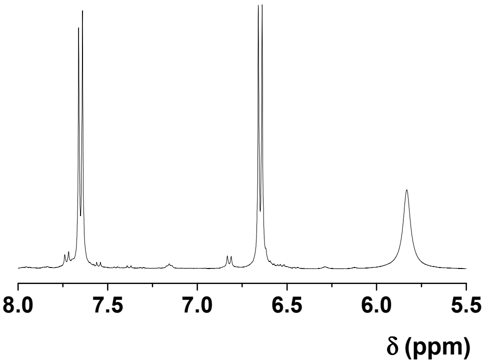

c)

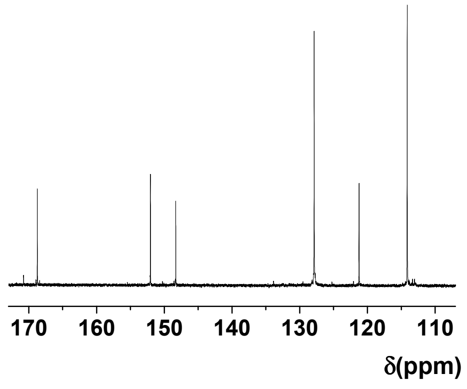

Figure S5. Characterization of **D2** by (a) FTIR, (b) ^{1}H NMR, and (c) ^{13}C NMR.

S3. Characterization of model polyamide M1

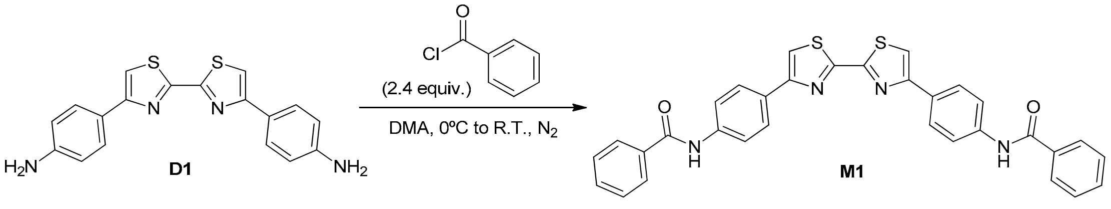

Characterization of N,N'-([2,2'-bithiazole]-4,4'diylbis(4,1-phenylene))dibenzamide (M1):

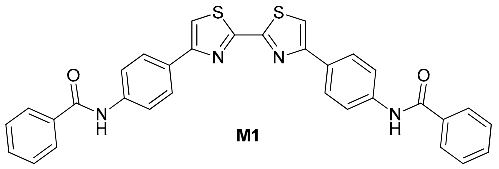

a)

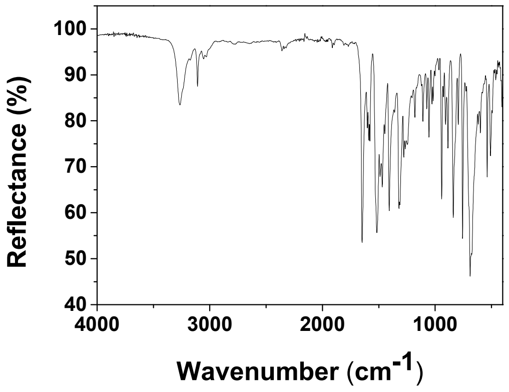

b)

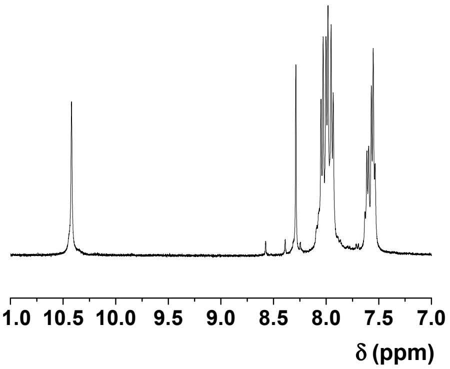

c)

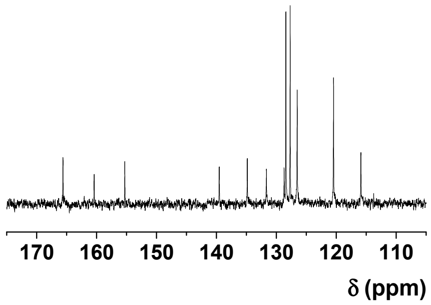

Figure S6. Characterization of **M1** by (a) FTIR, (b) ^{1}H NMR, and (c) ^{13}C NMR.

S4. Characterization of model polyamide M2

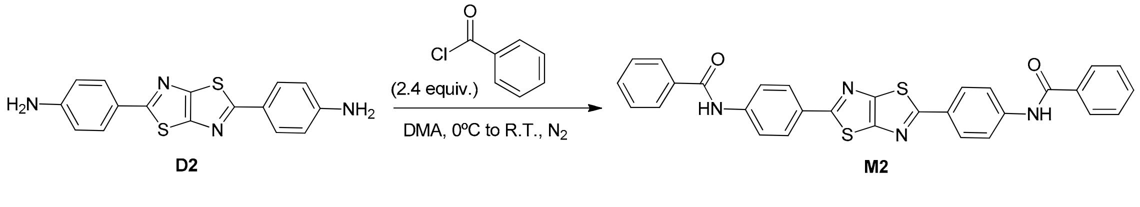

Characterization of N,N'-(thiazolo[5,4-d]thiazole-2,5-diylbis(4,1-phenylene)) dibenzamide (M2):

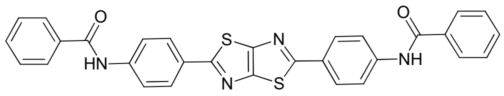

M2

a)

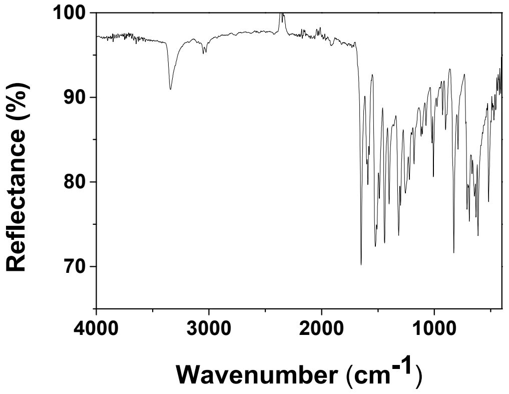

b)

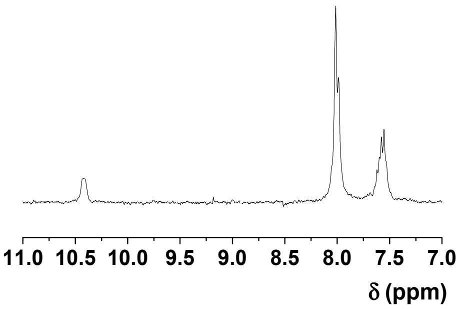

Figure S7. Characterization of **M2** by (a) FTIR, (b) ^{1}H NMR.

S5. Characterization of copolymers CP1 and CP2

Characterization of CP1:

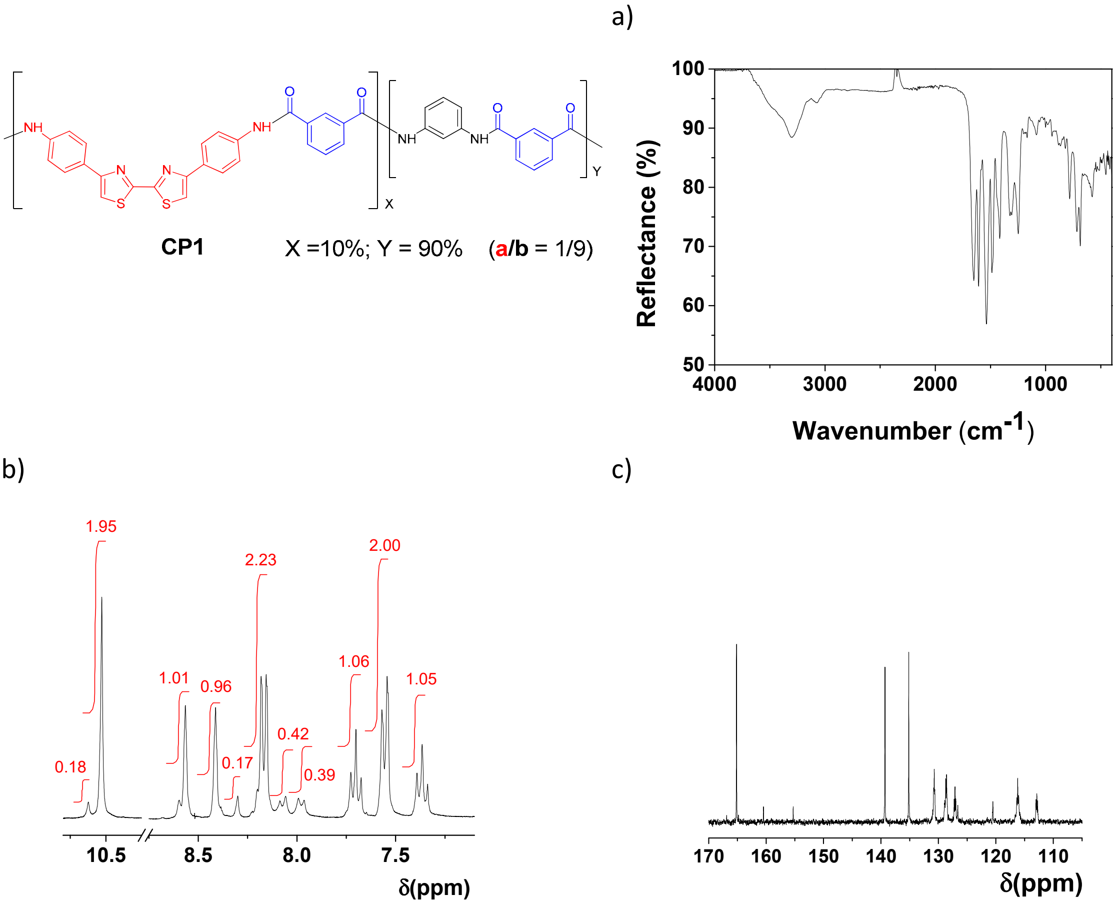

Figure S8. Characterization of **CP1** by (a) FTIR, (b) ^{1}H NMR, and (c) ^{13}C NMR.

Characterization of copolyamide CP2:

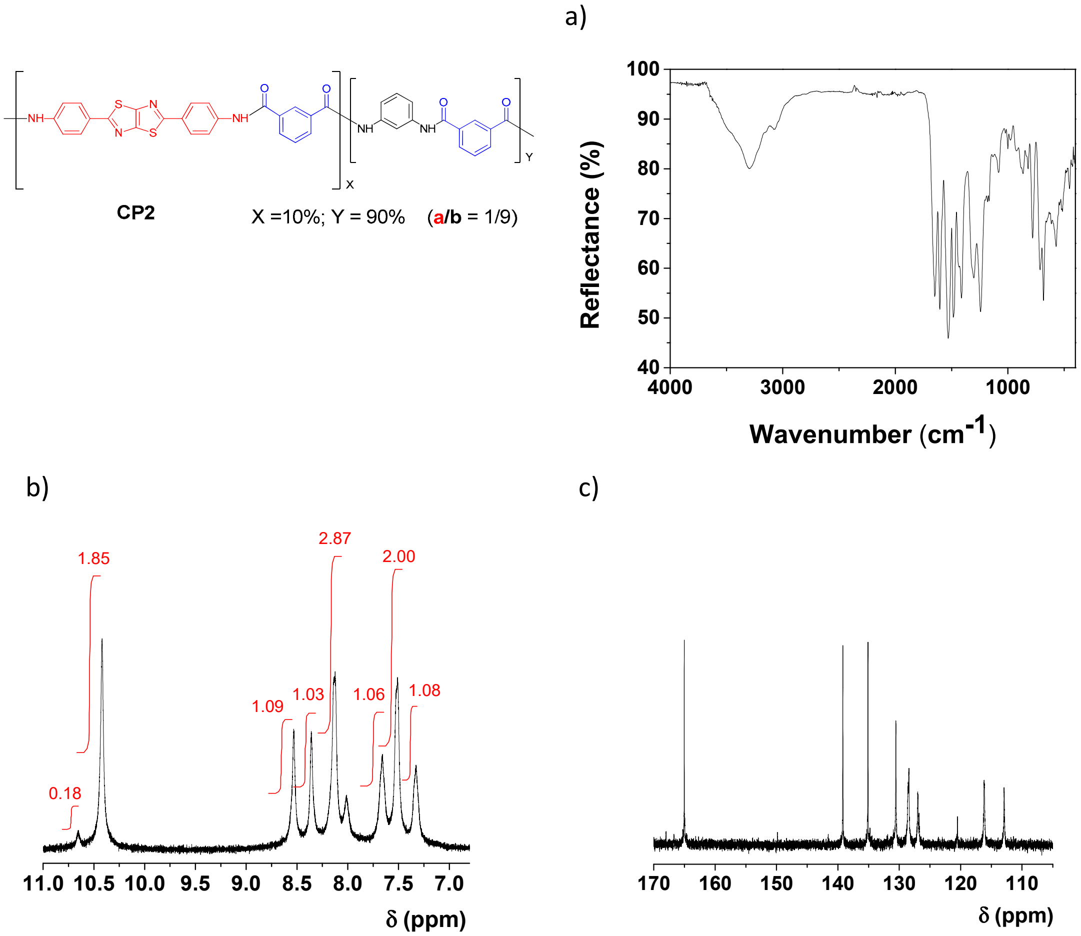

Figure S9. Characterization of **CP2** by (a) FTIR, (b) ^{1}H NMR, and (c) ^{13}C NMR.

Characterization of reference polyamide poly(m-phenylene isophthalamide):

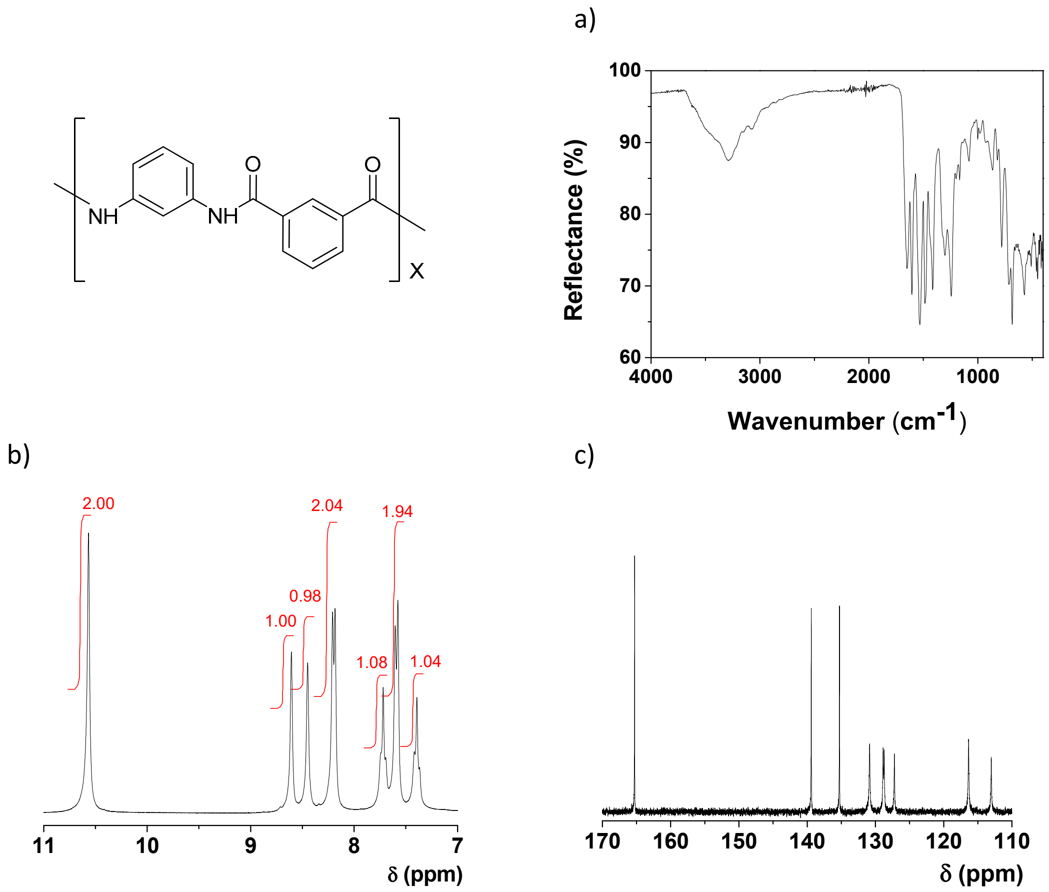

Figure S10. Characterization of reference polyamide poly(*m*-phenylene isophthalamide) by (a) FTIR, (b) ^{1}H NMR, and (c) ^{13}C NMR.

S6. Mechanical properties of polyamide films

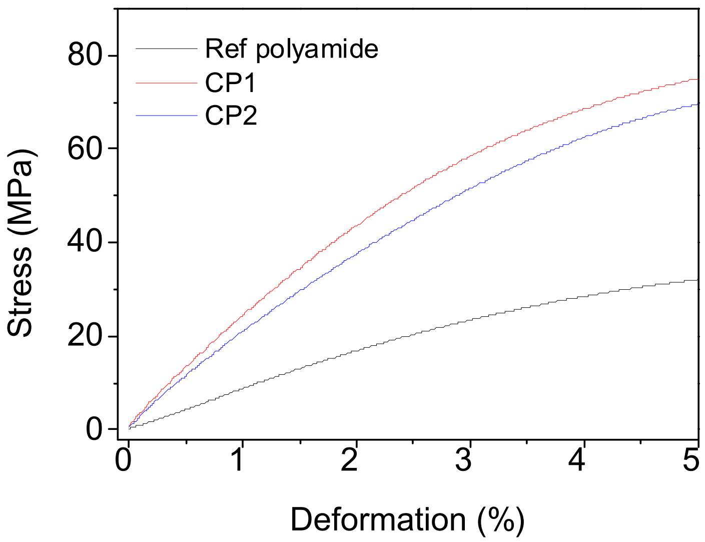

Figure S11. Representative stress-strain patterns of copolyamides **CP1**, **CP2**, and reference polyamide poly(*m*-phenylene isophthalamide). The graph has been enlarged at the initial deformation values to show the differences in the Young's Modulus.

S7. Thermogravimetric analysis of polyamides

a)

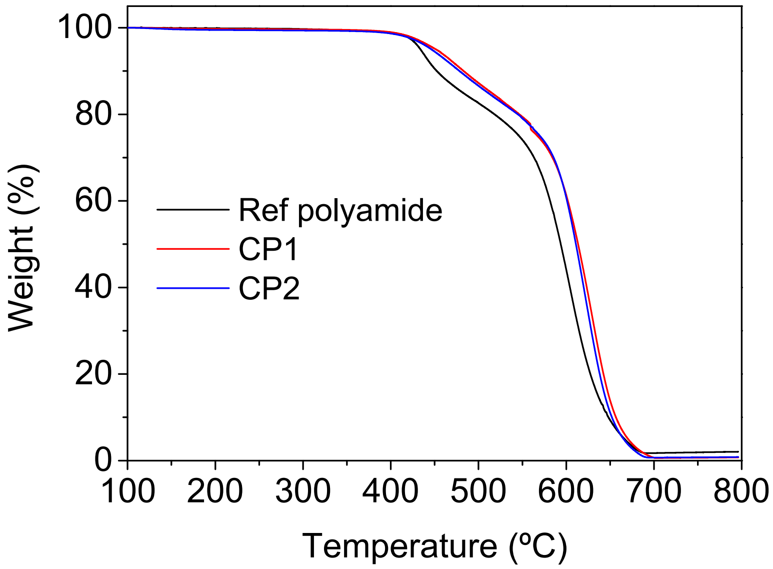

b)

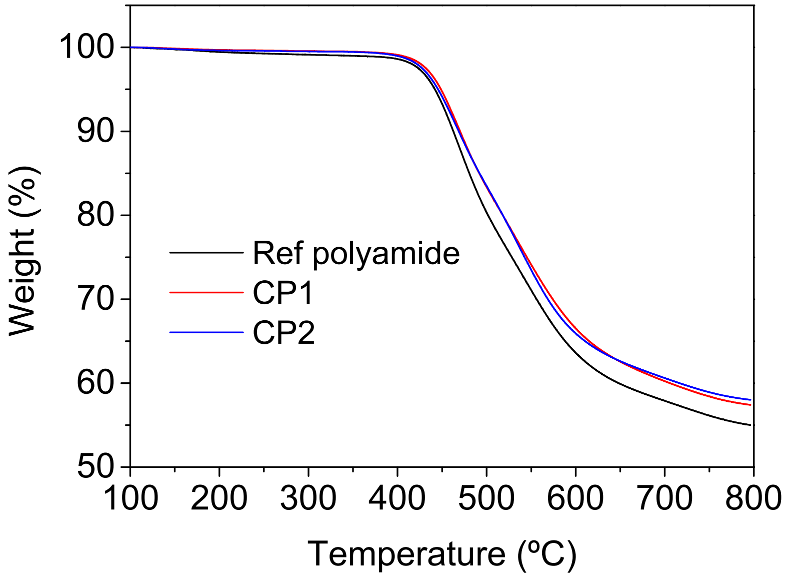

Figure S12. Thermogravimetric analysis of copolyamides **CP1**, **CP2**, and reference polyamide poly(*m*-phenylene isophthalamide) under a) synthetic air atmosphere, and b) nitrogen atmosphere.
